# Autoimmune thyroid disease and human health: a systematic review of Mendelian randomization studies

**DOI:** 10.3389/fimmu.2025.1689498

**Published:** 2025-12-04

**Authors:** Peijin Li, Qian Wang, Yan Yang, Zhiguo Ding

**Affiliations:** 1 Department of Thyropathy, Dongzhimen Hospital of Beijing University of Chinese Medicine, Beijing, China; 2 Department of Endocrinology, The Second People’s Hospital of China Three Gorges University, Yichang, Hubei, China; 3 Laboratory, Sunsimiao Hospital of Beijing University of Chinese Medicine, Tongchuan, Shanxi, China; 4 Hainan Medical University, Haikou, Hainan, China; 5 Thyroid Hospital, Sunsimiao Hospital of Beijing University of Chinese Medicine, Tongchuan, Shanxi, China; 6 Beijing University of Chinese Medicine, Beijing, China

**Keywords:** autoimmune thyroid disease, human health, systematic review, Mendelian randomization, Grave’s disease

## Abstract

We systematically summarized current Mendelian randomization (MR) evidence on the causal relationships between autoimmune thyroid disease (AITD) and a wide range of human health outcomes. Original MR studies related to AITD published up to March 1, 2025, were retrieved from PubMed and Embase. For studies investigating the same exposure–outcome associations, meta-analyses were performed to synthesize the evidence after excluding overlapping samples where applicable. The methodological quality of the included studies was assessed using the STROBE-MR checklist. A total of 123 MR publications met the inclusion criteria. MR analyses indicated that AITD significantly increased the risk of coronary atherosclerosis, deep venous thrombosis, chronic obstructive pulmonary disease, major depression, diabetic neuropathy, carpal tunnel syndrome, neuromyelitis optica spectrum disorder, diabetic retinopathy, childhood absence epilepsy, rheumatoid arthritis, systemic lupus erythematosus, psoriatic arthritis, inflammatory bowel disease, crohn's disease, vitiligo, myasthenia gravis, alopecia areata, type 1 and type 2 diabetes, sarcoidosis, frozen shoulder, drug eruptions, cataract, early age-related macular degeneration, pernicious anemia, Helicobacter pylori infection, temporomandibular disorders, migraine, frailty, primary biliary cholangitis, knee osteoarthritis, gout, and osteoporosis, while decreasing the risk of lung cancer, polyneuropathies, allergic rhinitis, telomere length, and serum vitamin A levels. The included MR studies were of high methodological quality and provided robust evidence supporting the bidirectional relationships between AITD and various health outcomes. These findings emphasize the importance and necessity of preventing and managing AITD while offering new perspectives and directions for future research on its prevention and treatment.

**Systematic review registration:**

https://www.crd.york.ac.uk/prospero/, identifier CRD42023469038.

## 1 Introduction

Autoimmune thyroid disease (AITD) is the most common organ-specific autoimmune disorder, primarily characterized by thyroid dysfunction and immune imbalance. Normal thyroid function is vital for growth, development, reproduction, and metabolism. As a major endocrine disorder, thyroid dysfunction disrupts glucose homeostasis, renal function, and reproductive health, posing significant health risks (1–3). Studies have shown that environmental factors such as radiation, smoking, and iodine intake, as well as certain endocrine disruptors like mercury and vanadium, are considered triggers of AITD (4). In addition, some comorbid conditions can further increase the risk of developing AITD (5). AITD primarily includes Graves’ disease (GD) and Hashimoto’s thyroiditis (HT). GD is the most frequent cause of hyperthyroidism in Western countries, predominantly affecting individuals aged 30 to 60 (6, 7). In contrast, HT is the leading cause of hypothyroidism in iodine-sufficient regions, with an overall prevalence of approximately 7.5% and a female prevalence rate of 17.5% (8). Studies indicate that AITD is associated with conditions such as vitiligo, alopecia areata, and celiac disease, as well as an increased risk of miscarriage and infertility in women (9–12). Additionally, it may contribute to various neuropsychiatric symptoms and alterations in brain function (13). Understanding the impact of AITD and its influencing factors is crucial for clinicians and researchers, aiding in developing personalized prevention and treatment strategies.

Mendelian randomization (MR) uses genetic variation as an instrumental variable to analyze causal relationships between exposures and outcomes. Because alleles of genetic variants are randomly assigned, conclusions can be made without randomized controlled trials, which can be limited by time, cost, and ethics (14). MR is increasingly popular as it better addresses confounding factors and reverse causality than observational studies. Genetic data related to exposures and outcomes, such as single-nucleotide polymorphisms (SNPs), can be obtained from genome-wide association studies (15) and screened according to the three core assumptions of MR: [1] instrumental variable is strongly associated with exposure, [2] instrumental variable is independent of any confounders affecting the exposure–outcome relationship, and [3] instrumental variable influences the outcome only through exposure and has no direct association with the outcome (16).

With MR advancements, numerous studies have analyzed the etiological factors associated with thyroid dysfunction. To date, one review has summarized the use of MR to assess the genetic correlations between thyroid disorders, including hyperthyroidism, hypothyroidism, and thyroid cancer, and various diseases (17). However, a comprehensive review specifically focused on AITD is still lacking. This study aimed to summarize and evaluate existing MR studies investigating AITD, including GD, HT, autoimmune hyperthyroidism (AIH), and autoimmune hypothyroidism (AIHT), as an exposure or outcome. These disease classifications are derived from varying definitions across relevant databases and involve distinct datasets. Furthermore, given that autoimmune thyroiditis and HT have the same disease definition, we will unify them in the study as HT. This study synthesizes evidence and identifies bidirectional causal factors of AITD, contributing to more effective prevention and treatment strategies.

## 2 Materials and methods

### 2.1 Search strategy and selection criteria

Data included in this study are publicly available, and ethics committee approval or patient informed consent was not required. This systematic review follows the Preferred Reporting Items for Systematic reviews and Meta-Analyses (PRISMA) (18) (**Supplementary Table S1**) and has been prospectively registered on the PROSPERO platform (registration number: CRD42023469038). Two independent investigators (PJL and QW) searched relevant literature from the inception of PubMed and Embase databases until March 1, 2025, using a combination of the search terms such as “thyroid dysfunction,” “thyroid disease,” “Hashimoto’s thyroiditis,” “Graves’ Disease,” “Autoimmune Thyroid Disease,” “autoimmune thyroiditis,” and “Mendelian randomization” (**Supplementary Table S2**). This study excluded reviews or meta-analysis through the search filters. In addition, conference abstracts, non-peer-reviewed articles, commentaries, and similar materials were also excluded. The retrieved literature was first imported into Zotero (https://www.zotero.org/) to remove duplicates, followed by title and abstract screening to exclude irrelevant studies. Full texts were reviewed to identify the final articles for inclusion. Additionally, manual reference checks of the included studies were conducted to avoid missing relevant studies. A list of the excluded studies along with the reasons for their exclusion is presented in **Supplementary Table S3**. In case of disagreement during literature search and selection, a third researcher (YY) was consulted for consensus. MR studies investigating AITD, including GD, HT, AIH, and AIHT, were included, without language or sample size restrictions.

### 2.2 Data extraction and quality assessment

Two independent researchers (PJL and YY) extracted the following information: exposure, outcomes, number of participants for each outcome, ancestry, number of SNPs used as genetic instrumental variables (IVs), and results of MR analysis. In addition, we summarized the multiple testing correction methods applied in the included studies, with detailed results provided in the **Supplementary Tables S4, S5**. In the absence of directional pleiotropy and heterogeneity between exposure and outcome, the inverse variance weighted (IVW) method yields the most accurate estimates (19). Due to space constraints, we primarily reported the IVW results in the manuscript. However, for each included study, we thoroughly examined all other analytical results (including weighted median, MR-Egger, and MR-PRESSO). Consistent findings across methods would indicate robust results, whereas inconsistencies might suggest potential pleiotropy. In such cases, we have explicitly stated and discussed these discrepancies. We evaluated the methodological quality of the included MR studies using the STROBE-MR checklist (20) (**Supplementary Table S6**), which is essential for improving the quality, transparency, and reproducibility of MR research. The checklist consists of 20 items across six domains. Each item was marked as “Y” (yes) if the criterion was fulfilled, “N” (no) if not fulfilled, or “NA” (not applicable). One reviewer (YY) independently performed the assessment, and another reviewer (QW) cross-checked the results. Any discrepancies were resolved through discussion with a third reviewer (ZGD).

### 2.3 Evaluation of evidence level and statistical analysis

We assessed the strength of causal evidence according to the criteria proposed by Chen et al. (21). If only the primary analysis (e.g., IVW) was performed, the MR association was rated as non-evaluable. For evaluable associations (those with a primary analysis and two sensitivity analyses), the evidence level was classified as high, moderate, low, very-low, or insufficient. When all methods produced statistically significant results with consistent effect directions, the evidence was rated as high quality; when the primary analysis and one sensitivity analysis yielded statistically significant results with consistent effect directions, the evidence was rated as moderate quality; when only one analysis showed a statistically significant result, the evidence was rated as low quality; and when none of the methods reached statistical significance, the evidence was rated as insufficient. If heterogeneity or horizontal pleiotropy was detected, the evidence level was downgraded by one grade. Among these, evidence classified as low quality would be downgraded by one level to very-low quality, rather than being regarded as insufficient evidence.

If studies with the same exposure–outcome relationship were available from at least two non-overlapping samples, a meta-analysis was performed using Review Manager 5.4 software. Possible sample overlap was carefully checked, and overlapping studies were not pooled in the meta-analysis. The *I^2^* statistic was used to assess heterogeneity between the studies. A random-effects model (DerSimonian–Laird estimator) was applied if heterogeneity was >50%. If significant heterogeneity exists (>90%), a meta-analysis was not be performed. Subgroup analyses were conducted based on disease types and population. Two independent researchers (QW and YY) contacted the corresponding authors for missing data. If data could not be retrieved, the study was excluded, and the impact of missing data was evaluated through sensitivity analysis. Additional sensitivity analyses were conducted to assess the influence of excluded low-quality studies, and result robustness was further tested using alternative effect models and leave-one-out analyses. Publication bias was assessed using funnel plots when over 10 studies were available for a given meta-analysis. To control for multiple comparisons across pooled associations, the false discovery rate (FDR) correction (Benjamini–Hochberg method) was applied at the meta-synthesis level, with an adjusted significance threshold of FDR < 0.05. For studies not included in the meta-analysis due to sample overlap or excessive heterogeneity, results were narratively summarized following the Synthesis Without Meta-analysis (SWiM) guideline (22) (**Supplementary Table S7**).

## 3 Results

### 3.1 Literature search results

**Figure 1:**
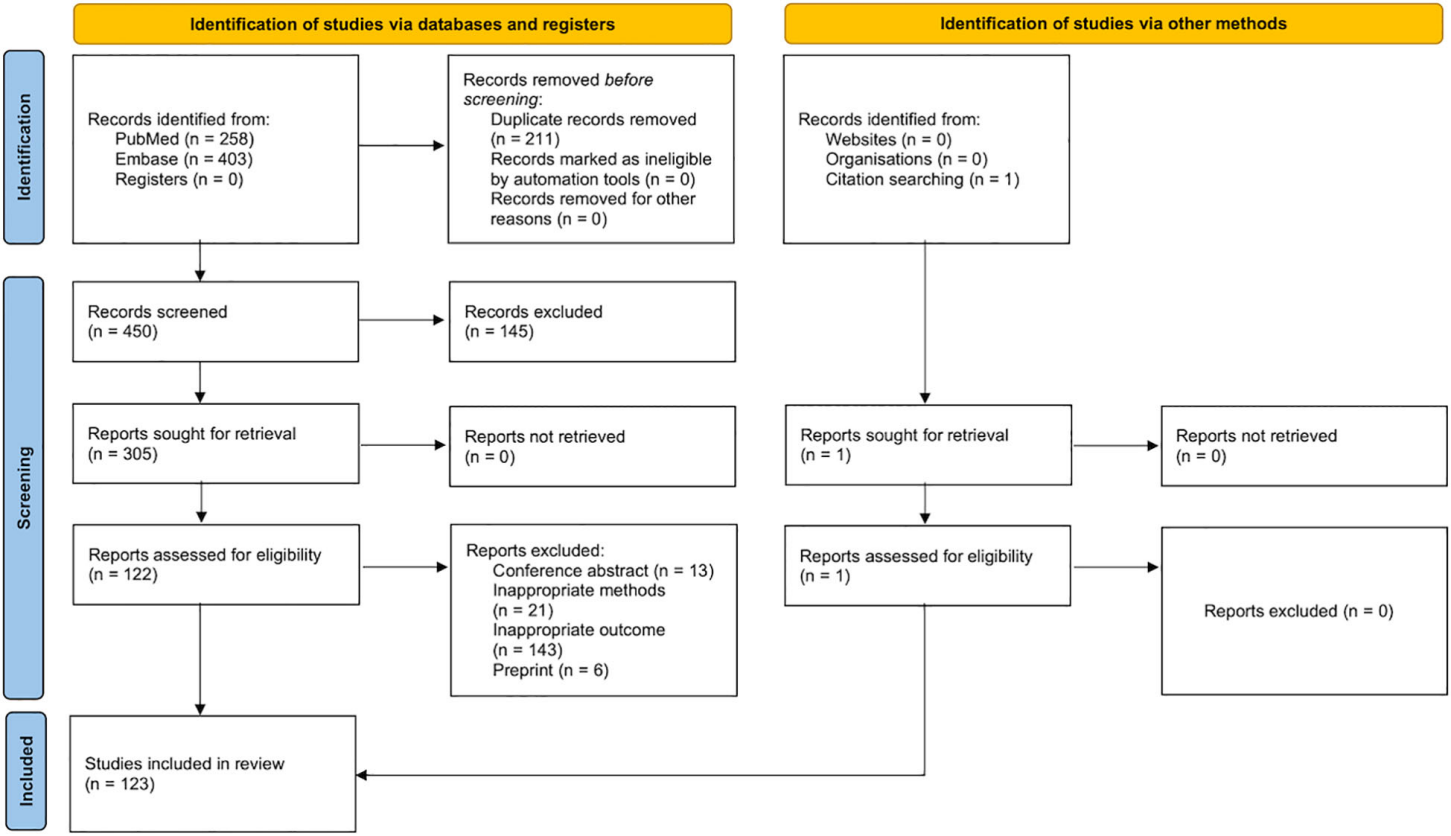
Study selection process.

The search yielded 662 publications. After removing duplicates (n = 211) and excluding irrelevant articles based on title and abstract screening (n = 145), 305 reports were identified. Following further exclusion of conference abstracts (n = 13), inappropriate methods (n = 21), inappropriate outcomes (n = 143), and preprints (n = 6), 123 eligible MR articles (23–145) were included (**Figure 1**). Notably, among the 143 studies excluded for inappropriate outcomes, 110 were removed due to unspecified autoimmune thyroid dysfunction.

### 3.2 Study description

Study characteristics are summarized in **Supplementary Tables S4, S5**. A total of 91 studies designated AITD as exposure, whereas 103 studies treated AITD as outcome. Publications spanned 2020-2025, with 73.98% appearing in 2024. When AITD served as exposure, studies collectively reported human health-related outcomes encompassing: 9 cardiovascular/respiratory outcomes, 12 oncological outcomes, 14 neuropsychiatric outcomes, 26 autoimmune outcomes, 4 hepatic outcomes, 6 Helicobacter pylori infection outcomes, 20 hematological outcomes, 7 ophthalmic outcomes, 5 otorhinolaryngological outcomes, 4 dermatological outcomes, 8 musculoskeletal/connective tissue outcomes, 3 senescence-related outcome, 5 miscellaneous pathological outcomes, 16 additional factor-related outcomes, and 23 outcomes related to blood/intestinal metabolites, alongside outcomes concerning gut microbiota, cytokines, and immune cells. When AITD was analyzed as outcome, studies identified 73 influencing factors for GD, 69 for HT, 47 for AIH, 67 for AIHT, and 14 for AITD. A total of 27 studies (28, 29, 48–50, 56, 60, 62, 64–66, 68, 69, 71, 74, 76, 78, 80, 83, 85, 115–121) conducted MR analyses on several identical exposure–outcome pairs. However, due to overlapping populations, a meta-analysis could not be performed. Instead, we synthesized the findings narratively in accordance with the SWiM guideline (**Supplementary Table S8**).

### 3.3 Quality assessment

Using STROBE-MR guidelines, 119 studies met ≥90% of criteria. All studies satisfied core methodological standards. In the study design and data sources section, two studies did not specify the eligibility criteria for participants, whereas 36 studies (29.27%) described the assessment methods for each exposure and outcome. Under main analysis, 45 studies (36.59%) conducted multiple tests. In the software and pre-registration section, 12 studies failed to clearly report the version of the statistical software used, and only one study had a pre-registered protocol. For additional analyses, 85 studies (69.11%) evaluated the direction of causality. In the discussion section, two studies did not address the limitations of research, and four studies omitted discussion on the mechanisms supporting causal effects. Moreover, none of the included studies exhibited evidence of weak instrument bias.

### 3.4 Cardiovascular diseases and respiratory diseases

Four publications (23–26) reported MR estimates for AITD and cardiovascular diseases (**Table 1**; **Supplementary Tables S4, S5**). Genetically predicted GD and HT showed no significant association with stroke or coronary artery disease. High evidence suggests that genetic liability to GD is associated with an increased risk of coronary atherosclerosis (odds ratio (OR), 1.04; 95% confidence interval (CI), 1.01-1.07; *P* = 0.002). Moderate evidence suggests that genetic liability to AIH is associated with an increased risk of deep vein thrombosis (OR, 1.00; *P* = 0.024). No significant causal association was observed between AIH, AIHT, and intracranial aneurysm. Six publications (27–32) reported MR estimates for AITD and respiratory diseases. Genetic liability to AIHT (OR, 1.00; *P* = 0.026) and GD (OR, 1.06; *P* = 0.020) is associated with an increased risk of chronic obstructive pulmonary disease (COPD). Very-low evidence suggests that genetic liability to COVID-19 is associated with an increased risk of AITD (**Supplementary Table S8**). Low evidence suggests that genetic liability to viral pneumonia is associated with an increased risk of AITD (OR, 1.10; *P* = 0.017).

**Table 1:** Summary of included MR studies for the effects of AITD on human health.

Human health ouctomes	Exposure: AITD with subtype				
	GD	HT	AIH	AIHT	AITD
Cardiovascular Diseases and Respiratory Diseases					
Coronary Atherosclerosis	OR = 1.04;95% CI 1.01–1.07;*p* = 0.002^a^	/	/	/	/
Coronary Artery Disease	Independence^d^	Independence^d^	/	/	/
Stroke	Independence^d^	Independence^d^	/	/	/
Deep Venous Thrombosis	/	/	OR, 1.0009;*p* = 0.024^b^	/	/
Intracranial Aneurysm	/	/	Independence^d^	Independence^d^	/
COPD	OR, 1.055 (1.008, 1.103);*p* = 0.02^c^	/	/	OR, 1.0004 (1.0001, 1.0008);*p* = 0.026^b^	/
COVID-19 infection	/	/	/	/	Independence^d^
Hospitalized/Severe COVID-19	/	/	/	/	Independence^d^
Asthma	/	/	/	Independence^d^	/
Cancer					
Endometrial Cancer	/	/	/	Independence^d^	/
Thyroid Cancer	/	Independence^d^	/	Independence^d^	/
Breast Cancer	Independence^d^	/	/	/	/
Lung Cancer	/	/	Independence^d^	OR, 0.918 (0.859, 0.982),*p* = 0.013^c^	/
Lung Adenocarcinoma	/	/	/	OR, 0.893 (0.813, 0.981),*p* = 0.019^b^	/
Squamous Cell Lung Cancer	/	/	/	OR, 0.888 (0.797, 0.990),*p* = 0.032^d^	/
Esophageal Cancer(East Asian)	Independence^d^	/	/	/	/
Gastric Cancer(East Asian)	Independence^d^	/	/	/	/
Colorectal Cancer(East Asian)	Independence^d^	/	/	/	/
Hepatocellular Carcinoma(East Asian)	Independence^d^	/	/	/	/
Biliary Tract Cancer(East Asian)	Independence^d^	/	/	/	/
Pancreatic Carcinoma(East Asian)	Independence^d^	/	/	/	/
Mental and Neurological Disorders					
Anxiety Disorder	/	Independence^d^	/	/	/
Depression	/	Independence^d^	/	/	/
Bipolar Disorder	/	Independence^d^	/	/	/
Major Depression	/	Independence^d^	/	OR, 1.020 (1.004, 1.037),*p* = 0.015^b^	/
Postpartum Depression	Independence^n^	Independence^n^	/	/	/
Posttraumatic Stress Disorder	/	/	/	/	Independence^n^
Focal Epilepsy	/	/	/	/	Independence^d^
Juvenile Absence Epilepsy	/	/	/	/	Independence^d^
Generalized Epilepsy With Tonic-Clonic Seizures	/	/	/	/	Independence^d^
Juvenile Myoclonic Epilepsy	/	/	/	/	Independence^d^
Epilepsy, All Documented Cases	/	/	/	/	Independence^d^
Generalized Epilepsy, All Documented Cases	/	/	/	/	Independence^d^
Childhood Absence Epilepsy	/	/	/	/	β = 0.005,SE = 0.002, *p* = 0.046^d^
Alzheimer’s Disease	Independence^d^	/	/	/	/
Autoimmune Disease					
Rheumatoid Arthritis	European: OR, 1.10 (1.01, 1.18); *p* = 0.02^a^; East Asian: OR, 1.34 (1.21, 1.47); *p* = 2.33E × 10^-9 b^	OR, 2.45 (1.15, 5.25),*p* = 0.02^b^	Independence^c^	OR, 1.51 (1.37, 1.66); *p* = 1.10 × 10^-16 b^	OR, 1.43 (1.27, 1.60), *p* = 8.39 × 10^-10 n^
Systemic Sclerosis	Independence^d^	Independence^n^	/	Independence^d^	/
Systemic Lupus Erythematosus	European: Independence^d^; East Asian: OR, 1.21 (1.08, 1.35); *p* = 6.79 × 10^-4 b^	/	/	/	/
Psoriasis	/	Independence^d^	/	/	/
Psoriatic Arthritis	/	OR, 1.23 (1.08, 1.40);*p* = 0.00^b^	/	/	/
Vulgar Psoriasis /Guttate Psoriasis	Independence^d^	/	/	/	/
Sarcoidosis	/	/	/	OR, 1.13 (1.06, 1.21); *p* = 3.21 × 10^-2 c^	/
Amyotrophic Lateral Sclerosis	Independence^d^	/	/	/	/
Inflammatory Bowel Disease	OR, 1.24 (1.01, 1.52); *p* = 0.041^d^	Independence^d^	/	/	/
Crohn's Disease	OR, 1.30 (1.06~1.59); *p* = 0.01^d^	Independence^d^	/	/	/
Ulcerative Colitis	Independence^d^	Independence^d^	/	/	/
Myasthenia Gravis	OR, 1.31 (1.08, 1.60);*p* = 0.005^d^	/	Independence^d^	OR, 1.26 (1.08, 1.47);*p* = 0.002^d^	/
Celiac Disease	Independence^n^	/	/	Independence^n^	/
Neuromyelitis Optica Spectrum Disorder	/	/	/	/	OR, 13.56 (10.47, 16.65); *p* = 8.43 × 10^-18 a^
T1D	OR, 1.173 (1.117, 1.231); *p* = 1.913 × 10^-10 c^	/	/	/	/
T2D	OR, 1.059 (1.025,1.095); *p* = 0.001^c^	/	/	/	/
Background Diabetic Retinopathy	OR, 1.49 (1.38, 1.62); *p* = 2.7 × 10^-23 a^	/	/	/	/
Diabetic Retinopathy	OR, 1.26 (1.20, 1.32); *p* = 1.2 × 10^-2^4 ^a^	/	/	/	/
Nonproliferative Diabetic Retinopathy	OR, 1.5 (1.28, 1.76); *p* = 4.2 × 10^-7 b^	/	/	/	/
Proliferative Diabetic Retinopathy	OR, 1.16 (1.11, 1.21); *p* = 1.1 × 10^-10 a^	/	/	/	/
Vitiligo	Independence^d^	OR, 1.971 (1.201, 3.234); *p* = 0.007^c^	Independence^d^	/	/
Alopecia Areata	Independence^d^	OR, 1.396 (1.030,1.892); *p* = 0.031^a^	/	/	/
Diabetic Neuropathy	/	/	/	/	OR, 9.93 × 10^8^ (3.2 × 10^5^, 3 × 10^13^);*p* = 88.28 × 10^-5 n^
Nerve Root/Plexus Disorder	/	/	/	/	Independence^n^
Carpal Tunnel Syndrome	/	/	/	/	OR, 13.79 (2.14, 88.63);*p* = 0.006^n^
Polyneuropathies	/	/	/	/	OR, 1 × 10^-3^ (6.68 × 10^-5^, 0.02);*p* = 4.44 × 10^-5 n^
Blood and Intestinal Metabolites					
Glutamine	OR, 1.00 (0.99, 1.00); p = 0.028^c^	/	/	/	/
Stearate (18:0)	OR, 1.00 (1.00, 1.01); *p* = 0.049^b^	/	/	/	/
2-hydroxystearate	OR, 1.01 (1.00, 1.01); *p* = 0.047^c^	/	/	/	/
1-oleoylglycerol (1-monoolein)	OR, 1.02 (1.00, 1.03); *p* = 0.008^b^	/	/	/	/
2-hydroxyisobutyrate	OR, 1.00 (0.99, 1.01); *p* = 0.041^b^	/	/	/	/
3-(4-hydroxyphenyl)lactate	OR, 1.00 (0.99, 1.00); *p* = 0.043^c^	/	/	/	/
Scyllo-inositol	OR, 1.01 (1.00, 1.02); *p* = 0.046^a^	/	/	/	/
1-arachidonoylglycerophosphocholine	OR, 1.01 (1.00, 1.02); *p* = 0.007^b^	/	/	/	/
1-palmitoleoylglycerophosphocholine	OR, 1.01 (1.00, 1.02); *p* = 0.003^b^	/	/	/	/
1-docosahexaenoylglycerophosphocholine	OR, 1.01 (1.00, 1.02); *p* = 0.014^c^	/	/	/	/
N2,N2-dimethylguanosine	OR, 0.99 (0.99, 1.00); *p* = 0.049^c^	/	/	/	/
Glutaroyl carnitine	OR, 1.01 (1.00, 1.01); *p* = 0.044^b^	/	/	/	/
1-myristoylglycerophosphocholine	OR, 1.01 (1.00, 1.02); *p* = 0.029^c^	/	/	/	/
Phosphate	Independence^d^	/	/	/	/
Kynurenine	Independence^d^	/	/	/	/
Taurochenodeoxycholate	Independence^d^	/	/	/	/
Erythrose	OR, 0.99 (0.98, 1.00); *p* = 0.046^c^	/	/	/	/
Glycerol 2-phosphate	Independence^d^	/	/	/	/
3-methylxanthine	Independence^d^	/	/	/	/
4-ethylphenylsulfate	Independence^d^	/	/	/	/
4-androsten-3beta,17beta-diol disulfate 1	Independence^d^	/	/	/	/
4-androsten-3beta,17beta-diol disulfate 2	Independence^d^	/	/	/	/
Phenylalanylphenylalanine	Independence^d^	/	/	/	/
**Liver Diseases**					
Alcoholic Liver Disease	/	Independence^d^	/	/	Independence^d^
Non-Alcoholic Fatty Liver Disease	/	Independence	/	/	Independence^n^
Primary Biliary Cholangitis	Independence^n^	/	Independence^n^	OR, 1.10 (1.02, 1.20); *p* = 0.02^b^	Independence^d^
Primary Sclerosing Cholangitis	Independence^d^	Independence^d^	/	/	/
Helicobacter Pylori Infection					
Antibodies against H. pylori (CagA)	OR, 1.16 (1.07, 1.26); *p* = 2.1×10^-4 b^	Independence^d^	/	Independence^d^	/
Antibodies against H. pylori (OMP)	OR, 1.11 (1.06, 1.17); *p* = 3.2×10^-5 a^	Independence^d^	/	OR, 1.15 (1.09, 1.22); *p* = 1.4×10^-6 a^	/
Antibodies against H. pylori (IgG)	Independence^d^	Independence^d^	/	Independence^d^	/
Antibodies against H. pylori (UREA)	Independence^c^	Independence^d^	/	Independence^d^	/
Antibodies against H. pylori (VacA)	Independence^d^	Independence^d^	/	Independence^d^	/
Antibodies against H. pylori (Catalase)	Independence^d^	Independence^d^	/	Independence^d^	/
Hematologic Diseases and Indices					
Pernicious Anemia	/	/	/	/	OR, 1.343 (1.196, 1.507); *p* = 0.000^b^
RBC	/	/	/	/	Independence^d^
RDW	/	/	/	/	β, 0.022 (0.009, 0.034); *p* = 0.001^c^
HCT	/	/	/	/	Independence^d^
HGB	/	/	/	/	Independence^d^
MCHC	/	/	/	/	Independence^d^
MCH	/	/	/	/	Independence^d^
MCV	/	/	/	/	Independence^d^
Reticulocyte Percentage	/	/	/	/	Independence^d^
Reticulocyte Percentage (irn)	/	/	/	/	β, -0.012 (-0.024, -0.001); *p* = 0.038^b^
Reticulocyte Count	/	/	/	/	β, 0.000 (-0.001, 0.000); *p* = 0.012^b^
Reticulocyte Count (irn)	/	/	/	/	β, -0.015 (-0.027, -0.003); *p* = 0.011^b^
Immature Reticulocyte Fraction	/	/	/	/	Independence^d^
Immature Reticulocyte Fraction (irn)	/	/	/	/	Independence^d^
Mean Reticulocyte Volume	/	/	/	/	Independence^d^
Mean Reticulocyte Volume (irn)	/	/	/	/	β, 0.027 (0.010, 0.045); *p* = 0.002^b^
Direct Bilirubin (umol/L)	/	/	/	/	Independence^d^
Direct Bilirubin (irn)	/	/	/	/	Independence^d^
Total Bilirubin (umol/L)	/	/	/	/	Independence^d^
Total Bilirubin (irn)	/	/	/	/	Independence^d^
Ophthalmic Diseases					
Diabetic Retinopathy	/	/	/	/	OR, 1.10 (1.04, 1.15); *p* = 3×10^-3 b^
Cataract	/	/	/	/	OR, 1.05 (1.02, 1.08); *p* = 3×10^-3 a^
Early AMD	/	/	/	/	OR, 1.019 (1.052, 1.154); *p* = 4.44 × 10^-5 b^
Late AMD	/	/	/	/	Independence^d^
Glaucoma	/	/	/	/	Independence^d^
Senile Cataract	OR, 1.031 (1.002, 1.060);*p* = 0.033^b^	/	/	OR, 2.4 (1.42, 4.06);*p* = 1.12×10^-3 c^	/
Age-related Macular Degeneration	Independence^d^	/	/	/	/
Otorhinolaryngologic Diseases					
Acute Sinusitis	Independence^a^	Independence^a^	/	/	/
Chronic Sinusitis	Independence^a^	Independence^a^	/	Independence^d^	/
Allergic Rhinitis	OR, 0.995 (0.991, 0.999); *p* = 0.021^b^	/	/	/	/
Nasal Polyps	/	/	Independence^d^	/	/
Temporomandibular Disorders	OR, 1.06 (1.01, 1.10); *p* = 0.012^c^	Independence^d^	/	/	/
Dermatologic Diseases					
drug eruption	OR, 1.303 (1.119, 1.516); *p* < 0.001^b^	/	/	/	/
generalized drug eruption	OR, 1.170 (1.041, 1.315); *p* = 0.008^b^	/	/	/	/
localized drug eruption	Independence^d^	/	/	/	/
Frozen shoulder	/	/	/	OR, 1.07 (1.01, 1.14); *p* = 0.02^a^	/
Musculoskeletal and Connective Tissue Disorders					
Fibromyalgia	/	/	/	Independence^d^	/
Osteoporosis	Independence^c^	/	Independence^d^	OR, 1.118 (1.068, 1.171); *p* = 0.000^b^	/
Osteoporosis With Pathological Fracture	OR, 1.131 (1.046, 1.224); *p* = 0.002^c^	/	Independence^c^	OR, 1.176 (1.083, 1.277); *p* = 0.000^a^	/
Postmenopausal Osteoporosis With Pathological Fracture	OR, 1.138 (1.064, 1.240); *p* = 0.003^c^	/	Independence^c^	Independence^c^	/
Knee Osteoarthritis	/	/	OR, 1.05 (1.03, 1.07); *p* = 0.000^b^	/	/
Hip Osteoarthritis	/	/	Independence^d^	/	/
Sarcopenia	OR, 1.027 (1.009, 1.046);*p* = 0.004^b^	/	/	/	/
Gout	/	/	OR, 1.07 (1.01, 1.12); *p*_FDR_ = 0.0314^b^	OR, 1.13 (1.03, 1.24); *p*_FDR_ = 0.0336^c^	/
Aging-related Diseases and Indicators					
Frailty Index	/	/	OR, 1.024 (1.004, 1.045); *p* = 0.0163^c^	OR, 1.023 (1.008, 1.038); *p* = 0.0015^c^	/
Facial Ageing	Independence^d^	/	/	Independence^d^	/
Age-related Hearing Impairment	OR, 1.009 (1.004, 1.014); *p* = 0.001^b^	/	/	/	/
Other Diseases					
Primary Ovarian Insufficiency	/	/	/	/	Independence^d^
Anti-Müllerian Hormone Levels	/	/	/	/	Independence^d^
Migraine	/	/	OR, 1.066 (1.030, 1.103); *p* = 0.000^b^	Independence^d^	/
Migraine with Aura	/	/	OR, 1.074 (1.021, 1.131); *p* = 0.006^b^	Independence^d^	/
Migraine without Aura	/	/	OR, 1.068 (1.012, 1.127); *p* = 0.017^c^	OR, 1.076 (1.004, 1.152); *p* = 0.038^d^	/
Other Factors					
Vitamin D	Independence^n^	Independence^n^	Independence^d^	Independence^d^	/
Vitamin A	/	/	OR, 0.97 (0.95, 1.00); *p* = 0.044^a^	Independence^d^	/
Vitamin B9	/	/	Independence^d^	Independence^d^	/
Vitamin B12	/	/	Independence^d^	Independence^d^	/
Vitamin C	/	/	Independence^d^	Independence^d^	/
Vitamin E	/	/	Independence^d^	Independence^d^	/
Daytime Nap	Independence^d^	/	/	Independence^d^	/
Getting Up	Independence^d^	/	/	Independence^d^	/
Sleep Duration	Independence^d^	/	/	Independence^d^	/
Daytime Sleepiness	Independence^d^	/	/	Independence^d^	/
Short Sleep	Independence^d^	/	/	Independence^d^	/
Long Sleep	Independence^d^	/	/	Independence^d^	/
Insominia	Independence^d^	/	/	Independence^d^	/
Adulthood BMI	/	/	/	/	Independence^d^
Height	/	/	/	/	Independence^d^
Telomere Length	OR, 0.982 (0.969, 0.994);*p* = 0.004^b^	/	β, -1.93×10^-2^ (-2.85×10^-2^, -1.00×10^-2)^; *p* = 4.54×10^-5 b^	Independence^d^	/

a: high quality; b: moderate quality; c: low quality; d: very low quality or insufficient.

### 3.5 Cancer

Six publications (33–36) reported MR estimates for AITD and cancer (**Table 1**; **Supplementary Tables S4, S5**). Low evidence suggests that genetic liability to AIHT is associated with an decreased risk of lung cancer (OR, 0.92; 95% CI, 0.86-0.98; *P* = 0.013), especially lung adenocarcinoma, and no significant associations were observed with endometrial cancer or thyroid cancer. Genetically predicted HT showed no significant effects on thyroid cancer; however, low evidence suggests that genetic liability to thyroid cancer is associated with an increased risk of HT in European populations (OR, 1.08; 95% CI, 1.03-1.14; *P* = 0.001). For other cancer types, evidence to support an association between genetic liability to GD and breast cancer (in European populations) or digestive system cancers (in East Asian populations) is insufficient.

### 3.6 Mental and neurological disorders

There were 10 publications (32, 39–47) that reported MR estimates for AITD and mental and neurological disorders (**Table 1**; **Supplementary Tables S4, S5**). Genetically predicted HT demonstrated no significant causal effects on anxiety, depression, bipolar disorder, postpartum depression, or major depression (MDD). Moderate evidence suggests that genetic liability to AIHT is associated with an increased risk of MDD (OR, 1.02; 95% CI, 1.00-1.04; *P* = 0.015). Low to moderate evidence suggests that genetically predicted depression (OR, 1.61; 95% CI, 1.10-2.36; *P* = 0.013), MDD (OR, 1.63; 95% CI, 1.29-2.05; *P* = 3.97×10^−5^) and postpartum depression (OR, 1.21; 95% CI, 1.09-1.34; *P* = 0.000) increased the risk of HT in European populations. These findings suggest distinct causal relationships between different depression phenotypes and AITD. Genetically predicted posttraumatic stress disorder increased the risk of AITD (β, 0.150; *P* = 0.0001) and GD (OR, 1.06; 95% CI, 1.01-1.11; *P* = 0.021).

### 3.7 Autoimmune disease

A total of 31 publications (48–78) reported MR estimates for AITD and other autoimmune diseases (**Table 1**; **Supplementary Tables S4, S5**). Genetically predicted HT increased the risk of rheumatoid arthritis (RA) (OR, 2.45; 95% CI, 1.15-5.25; *P* = 0.02), psoriatic arthritis (OR, 1.23; 95% CI, 1.08-1.40; *P* = 0.00), vitiligo (OR, 1.97; 95% CI, 1.20-3.23; *P* = 0.01), and alopecia areata (OR, 1.40; 95% CI, 1.03-1.89; *P* = 0.031). Three MR studies (48–50) examined the effects between GD and RA, and another three studies (50, 65, 76) examined the effects between GD and systemic lupus erythematosus (SLE). However, due to overlapping populations, these studies were not included in the meta-analysis for pooled estimation. Moderate to high evidence suggests that genetic liability to GD is associated with an increased risk of RA (European populations: OR, 1.10; 95% CI, 1.01-1.18; *P* = 0.02; East Asian populations: OR, 1.34; 95% CI, 1.21-1.47; *P* = 2.33×10^-9^) and SLE (East Asian populations: OR, 1.21; 95% CI, 1.08-1.35; *P* = 6.79×10^-4^) (**Supplementary Table S8**). Additionally, high evidence suggests that genetic liability to GD is associated with an increased risk of diabetic retinopathy (OR, 1.26; 95% CI, 1.20-1.32; *P* = 1.2×10^−24^) with subtypes. Low evidence suggests that GD is associated with an increased risk of type 1 diabetes (OR, 1.17; 95% CI, 1.12-1.23; P = 1.91 × 10-10) and type 2 diabetes (OR, 1.06; 95% CI, 1.03-1.10; *P* = 0.001). Very low evidence suggests that genetic liability to GD is associated with an increased risk of CD (OR, 1.30; 95% CI, 1.06-1.59; *P* = 0.01) in East Asian populations, inflammatory bowel disease (OR, 1.24; 95% CI, 1.01-1.52; *P* = 0.041), and myasthenia gravis (OR, 1.31; 95% CI, 1.08-1.60; *P* = 0.005). Genetically predicted AIHT was linked to increased risk of RA (OR, 1.51; 95% CI, 1.37-1.66; *P* = 1.10×10-16), sarcoidosis (OR, 1.13; 95% CI, 1.06-1.21; *P* = 0.032), and myasthenia gravis (OR, 1.26; 95% CI, 1.08-1.47; *P* = 0.002). High evidence suggests that genetic liability to AITD is associated with an increased risk of neuromyelitis optica (OR, 13.56; 95% CI, 10.47-16.65; *P* = 8.43×10^−18^). The results of the inverse MR analysis are presented in **Table 2**.

**Table 2:** Summary of included MR studies for the effects of human health on AITD.

Exposure: human health	AITD outcomes				
	GD	HT	AIH	AIHT	AITD
Cardiovascular Diseases and Respiratory Diseases					
Coronary Atherosclerosis	Independence^d^	/	/	/	/
Deep Venous Thrombosis	/	/	Independence^d^	/	/
Intracranial Aneurysm	/	/	Independence^d^	Independence^d^	/
COVID-19 infection	/	/	/	/	OR, 1.02 (1.00, 1.03); *p* = 0.037^d^
Hospitalized COVID-19	/	/	/	/	OR, 1.04 (1.01, 1.08); *p* = 0.012^d^
Severe COVID-19	Independence^d^	/	/	Independence^d^	OR, 1.06 (1.00, 1.12); *p* = 0.040^d^
Viral Pneumonia	/	/	/	/	OR, 1.10 (1.02, 1.18); *p* = 0.017^c^
Asthma (UKBB)	/	/	/	Independence^d^	/
Cancer					
Thyroid Cancer	/	European: OR, 1.0838 (1.0346, 1.1354); *p* = 0.001^b^; Asian: Independence^d^	/	Independence^d^	/
Endometrial Cancer	/	/	/	Independence^d^	/
Breast Cancer	Independence^d^	/	/	/	/
Lung Cancer	/	/	Independence^d^	Independence^d^	/
Mental and Neurological Disorders					
Anxiety Disorder	/	Independence^d^	/	/	/
Depression	/	OR, 1.614 (1.104, 2.358); *p* = 0.013^b^	/	/	/
Bipolar Disorder	/	Independence^d^	/	/	/
Borderline Personality Disorder	/	Independence^d^	/	/	/
Posttraumatic Stress Disorder	OR, 1.056 (1.008, 1.105); *p* = 0.021^c^	Independence^d^	/	/	β, 0.150, SE = 0.018; *p* = 0.0001^n^
Major Depression	/	OR, 1.627 (1.290, 2.053); *p* = 3.97 × 10^-5^ European: OR, 1.627 (1.290, 2.053); *p* = 3.97 × 10^-5 b^; East Asian: Independence^d^	/	/	/
Postpartum Depression	Independence^n^	OR, 1.209 (1.092, 1.338); *p* = 0.000^c^	/	/	/
Autoimmune Disease					
Rheumatoid Arthritis	European: OR, 1.269 (1.127,1.429); *p* = 8.15×10^-5 b^; East Asian: OR, 1.39 (1.10, 1.75); *p* = 0.007^a^	OR, 1.21 (1.12, 1.30); *p* = 3.95×10^-7 b^	OR, 1.47 (1.25, 1.72); *p* = 1.85×10^-6 b^	OR, 1.12 (1.09-1.16); *p* = 1.42×10^-8 b^	OR, 1.10 (1.04, 1.16); *p* = 6.53×10^-4 n^
Systemic Sclerosis	Independence^n^	Independence^n^	Independence^d^	Independence^d^	/
Systemic Lupus Erythematosus	European: Independence^d^; East Asian: OR, 1.10 (1.03, 1.17); *p* = 2.59×10^-3 c^	OR = 1.14 (1.04-1.26), *p* = 0.037^c^	Independence^d^	Independence^d^	/
Psoriasis	/	Independence^d^	Independence^d^	Independence^d^	/
Psoriatic Arthritis	/	Independence^d^	/	/	/
Vulgar Psoriasis /Guttate Psoriasis	Independence^b^	/	Independence^d^	Independence^d^	/
Generalilzed Pustular Psoriasis	Independence^a^	/	/	/	/
Sarcoidosis	/	/	/	Independence^c^	/
Amyotrophic Lateral Sclerosis	Independence^d^	/	/	/	/
Inflammatory Bowel Disease	Independence^d^	Independence^d^	/	/	/
Crohn's Disease	European: Independence^d^; East Asian: OR, 1.33 (1.15, 1.53); *p* < 0.001^b^	OR, 2.371 (1.526, 3.683); *p* < 0.001^b^	/	/	/
Ulcerative Colitis	European: OR, 1.09 (1.02, 1.16); *p* = 0.01^d^; East Asian: Independence^d^	Independence^d^	/	/	/
Myasthenia Gravis	OR, 1.50 (1.04, 1.98);*p* = 0.004^c^	/	OR, 1.55 (1.15, 2.09); *p* = 0.004^n^	OR, 1.29 (1.04, 1.59);*p* = 0.019^c^	/
Ankylosing Spondylitis	/	Independence^d^	/	/	/
Sjogren'S Syndrome	/	Independence^d^	/	/	/
Multiple Sclerosis	/	Independence^d^	/	/	/
Celiac Disease	OR, 1.25 (1.15, 1.36); *p* = 4.41 × 10^-8 a^	/	/	Independence^n^	/
Neuromyelitis Optica Spectrum Disorder	/	/	/	/	OR, 1.01 (1.00,1.01); *p* = 2.37 × 10^-3 c^
T1D	OR, 1.173 (1.117,1.231); *p* = 1.913 × 10^-10 b^	OR, 1.27 (1.11, 1.46); *p* < 0.001^d^	/	/	/
T2D	Independence^d^	/	/	/	/
Vitiligo	OR, 1.13 (1.06, 1.20); *p* < 0.01^b^	OR, 1.17 (1.01, 1.35); *p* = 0.04^c^	OR, 1.12 (1.03, 1.22); *p* = 0.01^b^	OR, 1.19 (1.11, 1.26); *p* = 1.12e × 10^-7 c^	/
Alopecia Areata	Independence^d^	Independence^d^	Independence^c^	Independence^d^	/
Pemphigus	/	/	Independence^d^	Independence^d^	/
Bullous Pemphigoid	/	/	Independence^d^	Independence^d^	/
Localized Scleroderma	/	/	Independence^d^	Independence^d^	/
Blood and Intestinal Metabolites					
Phosphate	OR, 0.088 (0.009, 0.861); *p* = 0.037	/	/	/	/
Kynurenine	OR, 3.851 (1.781, 8.325); *p* = 0.001	OR, 1.663 (1.223, 2.262); *p* = 0.001	/	/	/
Taurochenodeoxycholate	OR, 0.713 (0.517, 0.984); *p* = 0.040	/	/	/	/
Erythrose	OR, 0.366 (0.155, 0.863); *p* = 0.022	/	/	/	/
Glycerol 2-phosphate	OR, 0.549 (0.314, 0.961); *p* = 0.036	/	/	/	/
3-methylxanthine	OR, 0.576 (0.351, 0.945); *p* = 0.029	/	/	/	/
4-ethylphenylsulfate	OR, 0.580 (0.361, 0.931); *p* = 0.024	/	/	/	/
4-androsten-3beta,17beta-diol disulfate 1	OR, 0.746 (0.572; 0.974); *p* = 0.031	/	/	/	/
4-androsten-3beta,17beta-diol disulfate 2	OR, 0.461 (0.250, 0.851); *p* = 0.013	/	/	/	/
Phenylalanylphenylalanine	OR, 3.658 (1.098, 12.187); *p* = 0.035	/	/	/	/
4-acetamidobutanoate	OR, 0.28 (0.09, 0.84); *p* = 0.024^b^	/	/	/	/
palmitoylcarnitine	OR, 0.22 (0.07, 0.65); *p* = 0.006^a^	/	/	/	/
estrone 3-sulfate	OR, 0.82 (0.70, 0.96); *p* = 0.012^c^	/	/	/	/
3-methylhistidine	/	Independence^d^	/	/	/
Phenol Sulfate	/	Independence^d^	/	/	/
2-palmitoylglycerophosphocholine	/	Independence^d^	/	/	/
X-14189-leucylalanine	/	Independence^d^	/	/	/
Gamma-tocopherol	/	Independence^d^	/	/	/
Alpha-ketoglutarate	/	Independence^d^	/	/	/
Choline	/	OR, 1.795 (1.207, 2.671); *p* = 0.004^c^	/	/	/
Carotene diol (3)	/	OR, 0.694 (0.537, 0.896); *p* = 0.005^b^	/	/	/
Arachidonoylcholine	/	OR, 1.647 (1.187, 2.284); *p* = 0.003^c^	/	/	/
X-15486		OR, 1.721 (1.148, 2.578); *p* = 0.009^c^			
Glycolithocholate sulfate	/	OR, 1.341 (1.133, 1.587); *p* = 0.001^a^	/	/	/
5alpha-androstan-3alpha,17beta-diol disulfate	/	OR, 1.078 (1.007, 1.155); *p* = 0.032^c^	/	/	/
Maleate	/	OR, 0.616 (0.429, 0.885); *p* = 0.009^a^	/	/	/
Vanillic Alcohol Sulfate	/	OR, 1.508 (1.148, 1.981); *p* = 0.003^c^	/	/	/
Sphingomyelin	/	OR, 1.500 (1.107, 2.033); *p* = 0.009^c^	/	/	/
Theophylline	/	OR, 0.873 (0.789, 0.966); *p* = 0.008^b^	/	/	/
Trigonelline	/	OR, 0.908 (0.845, 0.976); *p* = 0.009^a^	/	/	/
10-nonadecenoate (19:1n9)	/	OR, 0.839 (0.739, 0.953); *p* = 0.007^b^	/	/	/
Piperine	/	OR, 1.134 (1.033, 1.244); *p* = 0.008^c^	/	/	/
S-methylcysteine sulfoxide	/	OR, 0.873 (0.796, 0.958); *p* = 0.004^b^	/	/	/
3-formylindole	/	OR, 0.915 (0.861, 0.972); *p* = 0.004^c^	/	/	/
Cholate	/	OR, 1.176 (1.059, 1.307); *p* = 0.002^b^	/	/	/
Nicotinamide	/	OR, 1.186 (1.082, 1.300); *p* = 0.000^b^	/	/	/
Arginine to glutamate ratio	/	OR, 1.151 (1.047, 1.264); *p* = 0.003^c^	/	/	/
3-methyl-2-oxovalerate to 4-methyl-2-oxopentanoate ratio	/	OR, 0.884 (0.791, 0.987); *p* = 0.029^c^	/	/	/
Indolelactate	/	/	/	OR, 1.592; *p_FDR_* = 0.036^c^	/
N- (3-furoyl)glycine	/	/	/	Independence^c^	/
Pipecolate	/	/	/	Independence^c^	/
Alanine	/	/	/	Independence^c^	/
Phenylalanine	/	/	/	Independence^c^	/
Allantoin	/	/	/	Independence^c^	/
Liver Diseases					
Primary Biliary Cholangitis	OR, 1.11 (1.06, 1.16); *p* = 0.000^a^	/	OR, 1.19 (1.12, 1.26); *p* = 0.000^a^	OR, 1.10 (1.08, 1.12); *p* = 0.000^a^	OR, 1.14 (1.12, 1.16); *p* = 0.000^a^
Primary Sclerosing Cholangitis	OR, 1.230 (1.089, 1.389); *p* = 0.001^b^	Independence^d^	/	/	/
Helicobacter Pylori Infection					
Antibodies against H. pylori (CagA)	Independence^d^	Independence^d^	/	Independence^d^	/
Antibodies against H. pylori (OMP)	OR, 1.70 (1.46, 1.98); *p* = 7.2×10^-12 c^	Independence^b^	/	Independence^d^	/
Antibodies against H. pylori (IgG)	Independence^d^	Independence^d^	/	Independence^d^	/
Antibodies against H. pylori (UREA)	Independence^d^	Independence^d^	/	Independence^c^	/
Antibodies against H. pylori (VacA)	Independence^d^	Independence^d^	/	Independence^d^	/
Antibodies against H. pylori (Catalase)	Independence^d^	Independence^d^	/	Independence^d^	/
Hematologic Diseases and Indices					
Pernicious Anemia	/	/	/	/	OR, 1.703 (1.378, 2.105); *p* = 0.000^c^
Ophthalmic Diseases					
Diabetic Retinopathy	/	/	/	/	OR, 1.34 (1.11, 1.62); *p* = 3×10^-4 c^
Cataract	/	/	/	/	Independence^d^
Senile Cataract	/	/	/	Independence^d^	/
Otorhinolaryngologic Diseases					
Chronic Sinusitis	/	/	/	Independence^d^	/
Nasal Polyps	/	/	Independence^d^	/	/
Temporomandibular Disorders	Independence^c^	Independence^d^	/	/	/
Dermatologic Diseases					
Frozen Shoulder	/	/	/	Independence^c^	/
Atopic Dermatitis	/	/	Independence^c^	OR, 1.055 (1.021, 1.091); *p* = 0.001^d^	/
Seborrheic Dermatitis	/	/	Independence^d^	Independence^d^	/
Acne	/	/	Independence^d^	Independence^d^	/
Rosacea	/	/	Independence^d^	Independence^d^	/
Urticaria	/	/	Independence^d^	Independence^d^	/
Musculoskeletal and Connective Tissue Disorders					
Fibromyalgia	/	/	/	OR, 1.024 (1.004, 1.043); *p* = 0.018^c^	/
Gout	/	/	Independence^d^	Independence^d^	/
Knee Osteoarthritis	/	/	Independence^d^	/	/
Hip Osteoarthritis	/	/	Independence^d^	/	/
Other Diseases					
Primary Ovarian Insufficiency	/	/	/	/	OR, 1.03 (1.01, 1.05);*p* = 0.015^b^
Other Factors					
Vitamin D	European:Independence^d^; Asian: OR, 1.71 (1.25, 2.33); *p* =0.001^a^	OR 0.499, (0.289, 0.860); *p* = 0.012^b^	Independence^d^	Independence^d^	/
Vitamin A	/	/	Independence^d^	Independence^d^	/
Vitamin B9	/	/	Independence^d^	Independence^d^	/
Vitamin B12	/	/	Independence^d^	Independence^d^	/
Vitamin C	Independence^d^	Independence^d^	Independence^d^	OR, 0.69 (0.58, 0.83); *p* < 0.001^b^	/
Vitamin E	/	/	Independence^d^	Independence^d^	/
Copper	OR, 1.183 (1.020, 1.372); *p* = 0.025^n^	Independence^n^	/	/	/
Iron	Independence^d^	Independence^d^	/	/	/
Zinc	Independence^n^	Independence^n^	/	/	/
Calcium	Independence^d^	Independence^d^	/	/	/
Selenium	Independence^d^	Independence^d^	Independence^d^	/	/
Daytime Nap	Independence^d^	/	/	Independence^d^	/
Getting Up	Independence^d^	/	/	OR, 0.59 (0.45, 0.78); *p* = 1.99 × 10^-4 c^	/
Sleep Duration	Independence^d^	/	/	Independence^d^	/
Daytime Sleepiness	Independence^d^	/	/	Independence^d^	/
Short Sleep	Independence^d^	/	/	Independence^d^	/
Long Sleep	Independence^d^	/	/	Independence^d^	/
Insominia	Independence^d^	/	/	Independence^d^	/
BMI	Independence^d^	OR, 3.071 (1.324, 7.118); *p* = 0.008^c^	/	OR, 1.31 (1.16, 1.48); *p* < 0.001^b^	/
Height	/	/	/	/	OR, 1.04 (1.02, 1.07); *p* = 1.99 × 10^-3 b^
Adulthood BMI	/	/	/	/	Independence^d^
Childhood BMI	/	/	/	/	Independence^d^
Fetal Birth Weight	/	/	/	/	Independence^d^
Hip Circumference	Independence^d^	/	/	OR, 1.32 (1.16, 1.50); *p* < 0.001^c^	/
Waist Circumference	OR, 1.72 (1.23, 2.41); *p* = 0.002^c^	/	/	OR, 1.42 (1.20, 1.67); *p* < 0.001^c^	/
Waist-to-hip Ratio	Independence^d^	/	/	Independence^d^	/
Telomere Length	/	/	OR, 0.49 (0.34, 0.72); *p* = 2.83 ×10^-4 b^	OR = 0.86 (0.77, 0.96); *p* = 7.46×10^-3 b^	/
Leukocyte Telomere Length	OR, 1.64 (1.23, 2.17); *p* = 2.27 × 10^-4 a^	/	/	/	/
Alcohol Consumption	OR, 0.57 (0.46, 0.70); *p* = 2.47 × 10^-7 a^	/	/	/	/
Proxied Glucosamine	/	OR, 2.47 (1.49, 4.08); *p* = 4.25×10^-4 a^	Independence^b^	/	/
Alpha-Adrenoceptor Blockers	/	Independence^n^	/	/	/
Angiotensin Converting Enzyme Inhibitors	/	Independence^n^	/	/	/
Aldosterone Antagonists	/	Independence^n^	/	/	/
Adrenergic Neuron Blockers	/	European: OR, 0.96 (0.93, 0.99); *p* = 0.008^n^; Asian: Independence^n^	/	/	/
Angiotensin-Ii Receptor Antagonists	/	Independence^n^	/	/	/
Beta-Adrenoceptor Blockers	/	Independence^n^	/	/	/
Calcium Channel Blockers	/	European: OR, 0.96 (0.95, 0.98); *p* = 3.51 × 10^-5 n^; Asian: OR, 0.28,(0.12, 0.66); *p* = 3.54 × 10^-3 n^	/	/	/
Loop Diuretics	/	European: OR, 0.94 (0.91, 0.97); *p* = 3.57 × 10^-5 n^; Asian: Independence^n^	/	/	/
Thiazide Diuretics	/	European: OR, 0.98 (0.97, 0.99); *p* = 3.83 × 10^-3 n^; Asian: Independence^n^	/	/	/
NO_2_	/	/	Independence^d^	OR, 1.373 (1.139, 1.656); *p* = 0.001^c^	/
NO_X_	/	/	Independence^d^	OR, 1.338 (1.105, 1.620); *p* = 0.003^d^	/
PM10	/	/	Independence^d^	Independence^d^	/
PM2.5	/	/	Independence^d^	Independence^a^	/
Cognitive Performance	/	/	Independence^d^	β, -0.18, SE = 0.58; *p* = 9.82 × 10^-6 b^	/
Educational Attainment	/	/	Independence^d^	β, -0.23,SE = 0.05; *p* = 5.00 × 10^-6 b^	/
Highest-Level Math Class Completed	/	/	Independence^d^	β, -0.16,SE = 0.041; *p* = 8.88 × 10^-5 b^	/
Self-Reported Math Ability	/	/	Independence^d^	Independence^d^	/
ApoB	Independence^n^	Independence^n^	/	Independence^n^	/
LDL	Independence^n^	Independence^n^	/	Independence^n^	/
TC	Independence^n^	Independence^n^	/	Independence^n^	/
TG	Independence^n^	Independence^n^	/	Independence^n^	/
ApoB inhibition	/	OR, 0.462 (0.216,0.986);*p* = 0.046^n^	/	/	/
PCSK9 inhibition	OR = 0.551 (0.319,0.953);*p* = 0.033^n^	/	/	OR = 0.735 (0.598,0.903);*p* = 0.003^n^	/
LDLR inhibition	/	/	/	OR, 0.779 (0.624,0.972);*p* = 0.027^n^	/
NPC1L1 inhibition	/	/	/	OR, 0.599 (0.412,0.872);*p* = 0.007^n^	/
Mitochondrial DNA copy number	/	/	Independence^d^	OR, 1.133 (1.016, 1.262); *p* = 0.024^c^	/

a: high quality; b: moderate quality; c: low quality; d: very low quality or insufficient.

### 3.8 Gut microbiome

Eight publications (79–87) reported MR estimates for AITD and gut microbiome. There were 24 genera, three families, three orders, one class, and one phylum that showed a significant correlation with GD. Two MR studies (80, 83) examined the casual effects of genus Catenibacterium on GD; however, due to overlapping populations, these studies were not included in the meta-analysis for pooled estimation. Low evidence suggests that genetic liability to genus Catenibacterium is associated with an increased risk of GD (**Supplementary Table S8**). There were 13 genera, five families, two order, two classes, and two phylum that showed a significant correlation with HT. Two MR studies (83, 85) examined the casual effects of genus RuminococcaceaeUCG011 and genus Butyrivibrio on HT, and two studies (81, 85) examined the casual effects of family Alcaligenaceae on HT. These studies were not included in the meta-analysis for pooled estimation due to overlapping populations (**Supplementary Table S8**). Low to moderate evidence suggests that genetic liability to these exposures are associated with risk of HT. Four genera and two phylum showed a significant correlation with AIHT. Among these, high evidence suggests that genetic liability to phyla Actinobacteria is associated with an decreased risk of AIHT (OR, 0.827; 95% CI, 0.738-0.926; *P* = 0.001). Detailed results are presented in **Supplementary Tables S4, S5**.

### 3.9 Blood and gut metabolites

Five publications (84, 87–90) reported on the relationships between AITD and blood/gut metabolites (**Tables 1**, **2**). MR analyses revealed significant associations between 13 identified blood metabolites and GD risk, with kynurenine and phenylalanylphenylalanine being potential risk factors. A total of 21 genetically predicted blood metabolites were associated with HT risk, including 13 metabolites (arachidonoylcholine, kynurenine, piperine, choline, cholate, nicotinamide, arginine-to-glutamate ratio, 5α-androstan-3α,17β-diol disulfate, glycolithocholate sulfate, X-15486, vanillic alcohol sulfate, and sphingomyelin) showing risk-increasing effects, whereas 8 metabolites showed protective effects. Another study suggested seven additional blood metabolites potentially linked to HT, although none retained statistical significance after multiple testing correction. Furthermore, genetically predicted indolelactate, a gut-derived metabolite, was found to increase AIHT risk.

### 3.10 Other diseases

In liver diseases, genetically predicted HT showed no significant effects on alcoholic liver disease or non-alcoholic fatty liver disease (91). AIHT was associated with an increased risk of primary biliary cholangitis (OR, 1.10; 95% CI, 1.02-1.20; *P* = 0.02) (92). Reverse MR analysis suggested that primary sclerosing cholangitis increased the risk of GD (OR, 1.23; 95% CI, 1.09-1.39; *P* = 0.001) (93). Genetically predicted HT showed no significant association with Helicobacter pylori infection (94). However, GD was associated with elevated levels of antibodies against H. pylori cytotoxin-associated gene A (CagA) (OR, 1.16; 95% CI, 1.07-1.26; *P* = 2.1×10^−4^) and outer membrane protein (OMP) (OR, 1.11; 95% CI, 1.06-1.17; *P* = 3.2×10^−5^). AIHT also increased OMP antibody levels (OR, 1.15; 95% CI, 1.09-1.22; *P* = 1.4×10^−6^). Reverse MR analysis revealed that elevated OMP antibodies against H. pylori significantly increased the risk of GD after adjustment (OR, 1.70; 95% CI, 1.46-1.98; *P* = 7.2×10^−12^).

In hematologic diseases and indices, genetically predicted AITD was significantly associated with an increased risk of pernicious anemia (OR, 1.34; 95% CI, 1.20-1.51; *P* = 0.000) (95). AITD also showed positive genetic correlations with red cell distribution width, while exhibiting inverse associations with reticulocyte count. In ophthalmic diseases, genetically predicted AITD was significantly associated with diabetic retinopathy (OR, 1.10; 95% CI, 1.04-1.15; *P* = 3×10^−3^), cataract (OR, 1.05; 95% CI, 1.02-1.08; *P* = 3×10^−3^), and early age-related macular degeneration (AMD) (OR, 1.02; 95% CI, 1.05-1.15; *P* = 4.44×10^−5^), while showing no significant effects on late AMD or glaucoma (96). Separately, genetically predicted AIHT (OR, 2.4; 95% CI, 1.42-4.06; *P* = 1.12×10^−3^) and GD (OR, 1.03; 95% CI, 1.00-1.06; *P* = 0.033) both demonstrated significant associations with increased risk of senile cataract (32, 97).

In otorhinolaryngologic diseases, genetically predicted GD and HT showed no significant associations with acute or chronic sinusitis (98, 99). However, GD demonstrated a protective effect against allergic rhinitis (OR, 0.995; 95% CI, 0.991-0.999; *P* = 0.021) (100), while no significant relationship was observed between AIH and nasal polyps (101). Notably, GD was associated with increased risk of temporomandibular disorders (OR, 1.06; 95% CI, 1.01-1.10; *P* = 0.012) (102). Regarding dermatologic diseases, genetically predicted GD significantly elevated risks of drug eruption (OR, 1.303; 95% CI, 1.119-1.516; *P* < 0.001) (103). AIHT was associated with increased risk of frozen shoulder (OR, 1.07; 95% CI, 1.01-1.14; *P* = 0.02) (104). Reverse MR analysis revealed that atopic dermatitis increased the risk of AIHT (OR, 1.055; 95% CI, 1.021-1.091; *P* = 0.001) (58).

In musculoskeletal and connective tissue disorders, genetically predicted AIHT showed no significant effect on fibromyalgia (105) but increased risks of osteoporosis (OR, 1.12; 95% CI, 1.07-1.17; *P* = 0.000) (106). GD was associated with elevated risks of osteoporosis/postmenopausal osteoporosis with pathological fracture, and sarcopenia (OR, 1.03; 95% CI, 1.01-1.05; *P* = 0.004) (30, 104). Both AIH (OR, 1.07; 95% CI, 1.01-1.12; *P*_FDR_ = 0.0314) and AIHT (OR, 1.13; 95% CI, 1.03-1.24; *P*_FDR_ = 0.0336) showed significant associations with gout risk after multiple testing correction (107). AIH specifically increased knee osteoarthritis risk (OR, 1.05; 95% CI, 1.03-1.07; *P* = 0.000) without affecting hip osteoarthritis (108).

Genetically predicted GD and AIHT showed no significant effects on facial aging (109). However, GD was associated with an increased risk of age-related hearing impairment (OR, 1.01; 95% CI, 1.00-1.01; *P* = 0.001) (32). Both AIHT (OR, 1.02; 95% CI, 1.01-1.04; *P* = 0.0015) and AIH (OR, 1.02; 95% CI, 1.00-1.05; *P* = 0.0163) were significantly associated with higher frailty index (110). Additionally, genetically predicted AIH was found to increase migraine risk (OR, 1.07; 95% CI, 1.03-1.10; *P* = 0.000) (111), whereas AIHT only elevated the risk of migraine without aura (OR, 1.08; 95% CI, 1.00-1.15; *P* = 0.038). AITD showed no significant association with either primary ovarian insufficiency or anti-Müllerian hormone levels (112, 113).

### 3.11 Other factors

Eight publications (114–121) reported on the relationship between AITD and serum vitamin/micronutrients levels (**Tables 1**, **2**). Genetically predicted AIH was associated with reduced vitamin A levels (OR, 0.97; 95% CI, 0.95-1.00; *P* = 0.044). Three MR studies (115, 119, 120) examined the effects of Vitamin D on HT, and another four studies (115, 116, 118, 120) examined the effects of Vitamin D on GD. However, due to overlapping populations, these studies were not included in the meta-analysis for pooled estimation. High evidence suggests that genetic liability to Vitamin D is associated with an increased risk of GD in Asian populations (OR, 1.71; 95% CI, 1.25-2.33; *P* = 0.001), and low evidence suggests that Vitamin D is associated with an decreased risk of HT (OR, 0.50; 95% CI, 0.29-0.86; *P* = 0.012) (**Supplementary Table S8**). Vitamin C levels decreased AIHT risk (OR, 0.69; 95% CI, 0.58-0.83; *P* < 0.001). Additionally, micronutrients levels showed no significant effects on GD, HT, or AIH.

Genetically predicted AIH (β, −1.93×10^−2^; *P* = 0.013) and GD (OR, 0.982; 95% CI, 0.969-0.994; *P* = 0.004) were significantly associated with reduced telomere length (32, 122, 123). Reverse MR analysis demonstrated that longer telomere length conferred protection against both AIH (OR, 0.49; 95% CI, 0.34-0.72; *P* = 2.83×10^−4^) and AIHT (OR, 0.86; 95% CI, 0.77-0.96; *P* = 7.46×10^−3^). Notably, leukocyte telomere length was associated with increased GD risk (OR, 1.64; 95% CI, 1.23-2.17; *P* = 2.27×10^−4^) (124). Genetically predicted higher mitochondrial DNA copy number was associated with increased risk of AIHT (OR = 1.133; 95% CI, 1.016-1.262; *P* = 0.024) (125). Higher body mass index (BMI) increased risks of both HT (OR, 3.071; 95% CI, 1.324-7.118; *P* = 0.008) and AIHT (OR, 1.31; 95% CI, 1.16-1.48; *P* < 0.001), whereas height was associated with increased AITD risk (OR, 1.04; 95% CI, 1.02-1.07; *P* = 1.99×10^−3^) (126–128). Additionally, both hip circumference (OR, 1.32; 95% CI, 1.16-1.50; *P* < 0.001) and waist circumference (OR, 1.42; 95% CI, 1.20-1.67; *P* < 0.001) were significantly associated with increased AIHT risk. Waist circumference also increased GD risk (OR, 1.72; 95% CI, 1.23-2.41; *P* = 0.002), with all associations remaining statistically significant after adjustment (129).

A study examining lipid profiles and lipid-lowering drug targets in relation to AITD found that lipid measures showed no significant associations with GD, HT, or AIHT (130). However, genetically predicted apolipoprotein B (ApoB) inhibition was associated with reduced HT risk (OR = 0.462; 95% CI, 0.216-0.986; *P* = 0.046). Similarly, proprotein convertase subtilisin/kexin type 9 (PCSK9) inhibition demonstrated protective effects against both GD (OR = 0.551; 95% CI, 0.319-0.953; *P* = 0.033) and AIHT (OR = 0.735; 95% CI, 0.598-0.903; *P* = 0.003). Additional protective associations were observed for low-density lipoprotein (LDL) receptor inhibition and NPC1-like intracellular cholesterol transporter 1 (NPC1L1) inhibition with AIHT risk. Regarding antihypertensive drugs (131), genetic evidence suggested protective effects against HT for adrenergic neuron blockers (OR, 0.959; 95% CI, 0.930-0.989; *P* = 0.008), calcium channel blockers (European population: OR, 0.963; 95% CI, 0.946-0.981; *P* = 3.51×10^−5^; Asian population: OR, 0.283; 95% CI, 0.121-0.661; *P* = 3.54×10^−3^), loop diuretics (OR, 0.940; 95% CI, 0.912-0.968; *P* = 3.57×10^−5^), and thiazide diuretics (OR, 0.979; 95% CI, 0.965-0.993; *P* = 3.83×10^−3^).

The study demonstrated that genetically predicted GD and AIHT showed no significant associations with sleep characteristics, whereas reverse MR analysis revealed getting up as a potential protective factor against AIHT (OR, 0.59; 95% CI, 0.45-0.78; *P* = 1.99×10^−4^) (132). Additionally, alcohol consumption was associated with reduced GD risk (OR, 0.57; 95% CI, 0.46-0.70; *P* = 2.47×10^−7^) (133), whereas proxied glucosamine use increased HT risk (OR, 2.47; 95% CI, 1.49-4.08; *P* = 4.25×10^−4^) (134). One publication investigating air pollutants’ effects on AITD found that genetically predicted NO_2_ (OR, 1.373; 95% CI, 1.139-1.656; *P* = 0.001) and NOx (OR, 1.338; 95% CI, 1.105-1.620; *P* = 0.003) were significantly associated with increased AIHT risk (135). Another publication examining educational influences demonstrated that cognitive performance, educational attainment, and highest-level math class completed all showed protective associations against AIHT risk (136).

### 3.12 Inflammatory factors

Six publications (137–142) investigated the potential causal associations between AITD and cytokine profiles and genes (**Supplementary Tables S4, S5**). Cytokines are messenger proteins released by immune cells and are closely associated with AITD. Regarding GD, genetic analysis identified that the pro-inflammatory cytokine TNF-β and cell surface protein corneodesmosin (CDSN) increased GD risk, whereas stem cell growth factor-β (SCGF-β), epithelial discoidin domain-containing receptor 1 (DDR1), and major histocompatibility complex class I-related gene A (MICA) conferred protective effects against GD. In the analysis of HT in European populations, we found that pro-inflammatory cytokines such as IL-12p70, IFN-γ, and IL-13 were positively correlated with HT risk, whereas the chemokine CCL2 and pro-inflammatory cytokine TNF-α were negatively correlated with HT risk. Additionally, multiple genes, including chondroitin sulfate N-acetylgalactosaminyltransferase 1 (CSGALNACT1), glutamate receptor-interacting protein 1 (GRIP1), and T-cell receptor-associated transmembrane adapter 1 (TRAT1) were identified as potential risk factors for HT, and membrane-associated guanylate kinase inverted 3 (MAGI3), calcium/calmodulin-dependent protein kinase type IV (CAMK4), interleukin-7 receptor (IL7R), endoplasmic reticulum-to-nucleus signaling 1 (ERN1), and nucleotidyltransferase MB21D2 were identified as protective factors. After rigorous Bonferroni correction, genetically predicted growth inhibitory factor (GIF) and CDSN were identified as potential protective factors for AIHT, with robust and reliable results.

### 3.13 Immune cells

To systematically evaluate the role of immune cells in AITD, we summarized the MR results from four publications (90, 143–145) (**Supplementary Tables S4, S5**). Among 731 phenotypes encompassing immune cell counts, proportions, and surface protein expression levels, we identified multiple immune features with potential causal associations with HT. Genetic prediction supported associations between 36 immune cell phenotypes and increased HT risk, whereas 34 were associated with reduced risk of HT. The results demonstrated that aberrant activation and functional dysregulation of myeloid cells represent prominent features of HT. Among these, the activation of antigen-presenting cells, particularly elevated expression levels of human leukocyte antigen (HLA)-DR, a critical antigen presentation molecule, on dendritic cells (DC) and monocytes, emerged as a salient risk factor. This suggests that aberrant recognition and presentation of thyroid autoantigens may constitute an initiating factor in HT pathogenesis. The accumulation of myeloid-derived suppressor cells (MDSCs) was also significantly associated with HT risk. Regulatory T cell (Treg) subpopulations exhibited paradoxical patterns in HT, with certain activated Treg states showing positive correlations with HT risk, whereas certain quiescent Treg states demonstrated protective trends. Furthermore, specific B-cell phenotypes were also associated with HT risk; for instance, elevated autoantibody levels resulting from excessive B-cell receptor signaling activation represent a direct contributing factor to HT development.

## 4 Discussion

Over the past 5 years, over 300 MR studies have evaluated the causal relationships between AITD and diverse health outcomes. This systematic review comprehensively synthesizes this evidence, providing robust genetic support for the causal impact of AITD on multi-system health. Furthermore, multiple diseases and factors significantly influence the risk of AITD, as detailed in **Tables 1**, **2**.

Coronary atherosclerosis is the primary pathological process leading to coronary artery disease (CAD). MR studies have shown that genetic liability to GD is associated with an increased risk of coronary atherosclerosis but does not significantly affect CAD risk in European populations, suggesting that GD may primarily promote vascular inflammation and plaque formation rather than directly contributing to clinical CAD in these populations. Elevated thyroid hormone levels are associated with increased levels of various coagulation factors and fibrinogen (146), predisposing patients with hyperthyroidism to a hypercoagulable state, which may increase the risk of thrombotic events and plaque formation. An observational study in Asian populations reported that GD is a risk factor for CAD (147), highlighting that population-specific factors may influence disease susceptibility. Additionally, genetically predicted AITD subtypes have been found to increase the risk of COPD, which may be explained by the roles of cell-mediated and antibody-mediated autoimmunity in the pathogenesis of stable COPD (148).

AIHT is the leading cause of hypothyroidism in iodine-sufficient regions globally. Interestingly, low evidence suggests that genetic liability to AIHT is associated with an decreased risk of lung cancer, which may reflect enhanced immune surveillance under autoimmune conditions or metabolic changes associated with hypothyroidism. Previous observational studies have suggested that hypothyroidism may reduce the risk of various cancers, particularly in individuals over 60 years of age (149), potentially due to reduced oxidative stress and regulation of the phosphoinositide 3-kinase (PI3K)/protein kinase B (AKT) signaling pathway (150). Given its potential clinical significance, this finding warrants further investigation. Recent studies have found that various autoimmune diseases are associated with mental health conditions (151). A meta-analysis showed that patients with AITD have a higher likelihood of experiencing depressive and anxiety symptoms (152), although other studies have reported conflicting results (40), highlighting the need for MR studies to clarify potential causal relationships. Our study demonstrated that genetically predicted AIHT is a risk factor for major depression, whereas no significant associations were observed between HT and anxiety or depression overall, nor between GD, HT, and postpartum depression. These findings suggest that autoimmunity alone may be insufficient to drive the development of psychiatric symptoms and that thyroid dysfunction, particularly hypothyroidism, may represent the primary causal factor (153). This insight is of notable clinical relevance.

Although AITD is often described as a typical organ-specific autoimmune disease, in most patients, its disease burden extends well beyond the thyroid. Our findings support the presence of extensive overlap within the autoimmune disease network, consistent with previous observational (49, 154). This may reflect shared genetic susceptibility and immune pathways, as susceptibility genes for AITD such as forkhead box protein P3 (FOXP3) and cytotoxic T-lymphocyte protein 4 (CTLA-4) can affect central and peripheral immune tolerance, thereby increasing the risk of developing other autoimmune diseases, including rheumatoid arthritis and systemic lupus erythematosus (155). Additionally, immune dysregulation, autoantibody production, and the release of inflammatory mediators, along with environmental triggers such as infections and chronic stress, may contribute to disease development through multiple mechanisms (156). Moreover, T1D plays an important mediating role in the associations between AITD and diabetic retinopathy, cataracts, and early AMD. AITD may also increase the risk of pernicious anemia through autoimmune mechanisms, such as the production of anti-parietal cell and anti-intrinsic factor antibodies, leading to vitamin B12 deficiency and impaired erythropoiesis (157). Collectively, these findings highlight the critical role of shared autoimmune origins in the development of these comorbid conditions.

This study further demonstrates that genetic liability to AITD with its subtypes is associated with an increased or decreased risk of various other diseases, underscoring the importance of AITD prevention and management. Notably, moderate evidence suggests that genetic liability to GD is associated with an decreased risk of allergic rhinitis, a finding that contradicts previous research (158). The presence of heterogeneity may partly explain these inconsistencies, and further studies are warranted to validate this association. Conversely, genetic liability to GD is associated with an increased risk of drug eruptions, suggesting that impaired immune tolerance may increase susceptibility to certain adverse drug reactions by altering immune responses, highlighting the need for greater caution when prescribing medications to patients with GD. Our findings also revealed that genetic liability to AITD is associated with an increased risk of frozen shoulder, knee osteoarthritis, gout, osteoporosis, and migraine, consistent with previous observational studies (92, 106–108, 111). These associations suggest that shared immune mechanisms, including abnormal T-cell activation and cytokine dysregulation, may contribute to an elevated risk of musculoskeletal, neurological, and hepatobiliary diseases in patients with AITD. Importantly, genetic liability to both AIH and AIHT are associated with an increased frailty index, a clinical condition indicative of accelerated biological aging and associated with higher morbidity, mortality, and hospitalization rates (159). This suggests that thyroid autoimmunity and the resulting thyroid hormone imbalances may contribute to the progression of frailty, thereby accelerating biological aging.

There is a strong connection between the thyroid and the gut. The gut microbiota can regulate immune system function and inflammatory responses and can indirectly influence thyroid function through the absorption of essential minerals such as iodine and the modulation of neurotransmitters (160). Our MR analyses demonstrated significant genetically casual associations between AITD and specific gut microbiota, providing evidence supporting the thyroid–gut axis hypothesis and highlighting the potential of microbiome-based therapeutic targets for disease intervention. Additionally, the observed interactions between genetically predicted AITD and Helicobacter pylori infection further support the thyroid–gut axis hypothesis. OMP antibodies play a critical role in maintaining H. pylori density and colonization capacity, and genetically predicted OMP is associated with increased risk of GD, although clinical evidence remains limited. CagA antibodies, key virulence factors of H. pylori, were found to be elevated in association with GD but not HT, consistent with previous findings (161). These results suggest that distinct autoimmune mechanisms may influence the risk of H. pylori infection.

Our study also reported multiple cytokines, genes, and immune cell phenotypes that exert significant causal effects on GD, HT, and AIHT, further delineating the immune-related mechanisms underlying AITD pathogenesis from a genetic perspective. A particularly noteworthy finding was that certain genes exhibited opposing roles in different AITD subtypes. For example, CDSN, a protein highly expressed in the skin and hair follicles, was identified as a risk factor for GD but showed a protective effect in AIHT. This may provide molecular insight into the skin-specific manifestations of GD, such as pretibial myxedema, and suggests a potential connection between the skin–thyroid axis in autoimmune processes. In addition, IL-12p70, IFN-γ, and IL-13 were all identified as risk factors for HT, outlining an inflammatory milieu driven by both Th1 and Th2 immune responses. This finding is consistent with the pathological features of HT, where cellular and humoral immunity are jointly activated. Interestingly, several molecules traditionally considered proinflammatory, such as TNF-α and methyl-accepting chemotaxis protein I (MCP-1), demonstrated protective effects against HT in this study. This paradoxical observation may reflect a compensatory mechanism by which the immune system attempts to restore immunological homeostasis during disease progression.

The analysis of immune cell phenotypes revealed a close association between Treg dysfunction and the development of HT. Specifically, certain activated or cytokine-secreting Treg subsets (such as CD25 on CD39^+^ activated Tregs) were associated with an increased risk of HT, whereas resting Treg-related phenotypes (such as CD25 on CD39^+^ resting Tregs) exerted a protective effect. Moreover, our findings indicated that the activation of monocytes and myeloid cells was significantly correlated with HT risk. The upregulated expression of MHC class II molecules (HLA-DR) on various antigen-presenting cells (APCs), together with the expansion of inflammatory monocyte subsets and monocytic MDSCs, collectively pointed to a highly active antigen-presenting environment and a chronic inflammatory state, which may represent key driving forces in the pathogenesis and progression of HT. Additionally, the observed association between C-C chemokine receptor type 2 (CCR2) expression on monocytes and HT risk further underscores the recruitment of myeloid cells to inflammatory sites. Beyond these findings, alterations in the differentiation balance among T-cell subsets and the involvement of specific B-cell subpopulations also appear to contribute to the immunopathological processes underlying HT. In AIHT, the T-cell immunoreceptor with Ig and ITIM domains (TIGIT) and lymphocyte activation gene 3 protein (LAG3), along with the key chemokines C–X–C motif chemokine (CXCL) 9 and CXCL10 (which recruit Th1 cells) and CXCL13 (which recruits B cells and promotes lymphoid follicle formation), were all identified as risk factors, revealing a clearer picture of T/B-cell cooperative immune activation. Furthermore, Beta-2-microglobulin was identified as a risk factor, suggesting a crucial involvement of CD8^+^ T cell-mediated cytotoxicity in the pathogenesis of AIHT. This study also summarized the effects of genetically predicted telomere length, mitochondrial DNA copy number, and air pollutants on AITD, suggesting interactions among mitochondrial function, cellular aging, environmental factors, and thyroid autoimmunity.

Several modifiable factors have emerged as potential intervention targets for AITD. Previous observational studies have shown that essential vitamins and microelements involved in thyroid pathophysiology are associated with AITD risk (117, 162). Our study demonstrated that genetic liability to vitamin C and vitamin D levels is associated with a decreased risk of AITD subtypes, suggesting that vitamin supplementation may help prevent AITD by modulating immune function and reducing oxidative stress (163, 164), although further clinical studies are needed to confirm these findings. Additionally, genetic liability to lifestyle factors such as alcohol consumption, sleep characteristics, educational attainment, and obesity indicators were found to influence AITD risk, indicating that lifestyle modifications may contribute to prevention strategies. Given the close link between thyroid and lipid metabolism, our study did not find direct casual associations between lipid levels and AITD but identified potential protective effects of lipid-lowering medications, including ApoB and PCSK9 inhibitors, against AITD. The use of antihypertensive medications was also significantly associated with HT. These findings highlight the potential for drug repurposing in AITD prevention and treatment, warranting further investigation.

This study utilized MR to systematically synthesize evidence on the genetic liability to AITD and its associations with a wide range of health outcomes. Overall, these findings provide genetic-level support for potential links between AITD susceptibility and several extra-thyroid traits, suggesting that the genetic predisposition to AITD may have systemic implications beyond thyroid autoimmunity. These associations emphasize the importance of considering comorbid risk profiles when managing individuals with high AITD susceptibility. Furthermore, this MR synthesis identified multiple exposures that show genetic associations with increased AITD risk, offering preliminary insights into modifiable factors that could inform prevention or early intervention strategies. Overall, by leveraging genetic instruments and minimizing confounding, this study contributes methodologically robust evidence that complements conventional epidemiological findings and provides a foundation for future mechanistic and interventional research.

This study has several limitations. First, as a summary analysis of MR studies, it does not include patient-level data. Second, due to potential sample overlap and methodological heterogeneity among the included studies, quantitative meta-analysis could not be performed for all exposure–outcome pairs. Therefore, some results were synthesized narratively, which may reduce the statistical power to detect consistent associations. Third, most of the included studies were conducted in European populations, which may limit the generalizability. Finally, although MR studies are superior to traditional observational studies in inferring causality, they cannot determine the effects of interventions. Future studies should prioritize validation across diverse populations, elucidate the mechanisms underlying the identified exposure–outcome associations, conduct clinical trials based on modifiable risk factors, and explore opportunities for drug repurposing, such as the use of lipid-lowering agents.

## 5 Conclusion

Evidence from MR studies suggests that genetic liability to AITD is associated with a range of human health outcomes. This indicates that AITD susceptibility may share common biological pathways with multiple systemic disorders, underscoring the importance of integrated disease management and further mechanistic investigation. In addition, the identification of modifiable exposures genetically associated with AITD risk provides valuable insights into potential preventive strategies and therapeutic targets. While these findings enhance our understanding of the complex interplay between thyroid autoimmunity and other diseases, they should be interpreted with caution given the population and methodological limitations of the included studies. Further high-quality, large-scale MR analyses and randomized controlled trials are warranted to validate these associations and facilitate their translation into clinical practice.

## Data Availability

The original contributions presented in the study are included in the article/**Supplementary Material**. Further inquiries can be directed to the corresponding author.
